# The efficacy and tolerability of combining pemetrexed-based chemotherapy with gefitinib in the first-line treatment of non-small cell lung cancer with mutated EGFR: A pooled analysis of randomized clinical trials

**DOI:** 10.1371/journal.pone.0275919

**Published:** 2022-10-10

**Authors:** Bi-Cheng Wang, Wen-Xuan Zhang, Bo-Hua Kuang, Guo-He Lin

**Affiliations:** 1 Cancer Center, Union Hospital, Tongji Medical College, Huazhong University of Science and Technology, Wuhan, China; 2 Department of Oncology, the Second Affiliated Hospital of Anhui Medical University, Hefei, China; IRCCS Giovanni Paolo II Cancer Hospital, ITALY

## Abstract

**Background:**

Epidermal growth factor receptor (EGFR)-tyrosine kinase inhibitor (TKI) monotherapy is the standard of care in treating advanced non-small cell lung cancer (NSCLC). Nevertheless, whether adding pemetrexed-based chemotherapy to EGFR-TKI targeted therapy furtherly prolongs survival outcomes and improves responses remains controversial. Therefore, we conducted this pooled analysis to compare the efficacy and tolerability between gefitinib plus pemetrexed-based chemotherapy and gefitinib alone in the first-line treatment of advanced NSCLC patients with mutated EGFR.

**Methods:**

We systematically searched PubMed, Web of Science, Embase, and Cochrane CENTRAL on June 23, 2022. Eligible studies were registered randomized clinical trials comparing gefitinib plus pemetrexed-based chemotherapy with gefitinib alone. The primary outcomes were overall survival (OS) and progression-free survival (PFS). Objective response rate (ORR), disease control rate (DCR), and discontinuation rate (DR) were explored as secondary outcomes.

**Results:**

Eight studies within five randomized clinical trials were eligible. Gefitinib combined with pemetrexed-based chemotherapy significantly prolonged OS (hazard ratio [HR] 0.57, 95% confidence interval [CI] 0.37–0.89, p = 0.0125) and PFS (HR 0.52, 95% CI 0.39–0.70, p < 0.0001) versus gefitinib alone. In subgroup analysis, patients with EGFR exon 19 deletion and exon 21 L858R could benefit from the addition of pemetrexed-based chemotherapy to gefitinib in terms of PFS (EGFR exon 19 deletion: HR 0.50, 95% CI 0.34–0.75, p = 0.0008; EGFR exon 21 L858R: HR 0.46, 95% CI 0.26–0.82, p = 0.0079) but not OS. In addition, ORR was improved after the administration of gefitinib plus pemetrexed-based chemotherapy against gefitinib (odds ratio [OR] 1.91, 95% CI 1.44–2.55, p < 0.0001). Both strategies showed comparable DCRs (OR 1.46, 95% CI 0.94–2.26, p = 0.0952) and DRs (risk ratio [RR] 2.80, 95% CI 0.69–11.44, p = 0.1509).

**Conclusion:**

Compared with gefitinib alone, combining pemetrexed-based chemotherapy with gefitinib significantly improved OS and PFS in advanced EGFR-mutant NSCLC patients with acceptable tolerability. However, the accurate sub-population who could have OS benefits requires further validation.

## Introduction

Chemotherapy, targeted therapy, and immunotherapy have been applied in the front-line treatments of lung cancer [1, 2]. For advanced non-small cell lung cancer (NSCLC) patients harboring mutated epidermal growth factor receptor (EGFR) (especially exon 19 deletion and exon 21 L858R), first-generation small molecule tyrosine kinase inhibitors (TKIs) could be the first-line option [3]. Although EGFR TKIs have been certificated to be superior to standard chemotherapy by numerous clinical trials [4–6], drug resistance may develop within 8–12 months [7].

In terms of gefitinib, the addition of pemetrexed-based chemotherapy might be an effective strategy that could enhance efficacy and decrease resistance. In JMIT trial, concurrent pemetrexed and gefitinib were administered in advanced non-squamous NSCLC patients with EGFR exon 19 deletion and exon 21 L858R [8]. Final results indicated that gefitinib combined with pemetrexed significantly improved progression-free survival (PFS) (16.2 months vs. 11.1 months) and numerically longer overall survival (OS) (43.4 months vs. 36.8 months) [9]. However, in another trial reported by Yang’s team, gefitinib combined with pemetrexed and cisplatin failed to prolong OS versus gefitinib alone (32.4 months vs. 45.7 months) in EGFR-mutated NSCLC patients [10]. The combination of gefitinib and pemetrexed-based chemotherapy in the first-line treatment of advanced NSCLC patients with mutated EGFR is still debatable.

Previously published meta-analyses had been conducted to explore the optimal first-line treatment for NSCLC harboring EGFR mutations and suggested gefitinib plus pemetrexed-based chemotherapy as one of the most effective strategies (the other one was osimertinib) [11, 12]. However, the enrolled data were published before 2018, and the final data were absent.

Updated results and newly designed clinical trials have been reported for the last three years. Therefore, we performed this pooled analysis of randomized clinical trials to assess the efficacy and tolerability of adding pemetrexed-based chemotherapy to gefitinib as the first-line treatment for advanced patients with EGFR-mutant NSCLC.

## Methods

### Search methods

We conducted this study according to the Preferred Reporting Items for Systematic Reviews and Meta-analyses (PRISMA) guideline [13]. A comprehensive search of prospective clinical trials was performed in PubMed, Web of Science, Embase, and Cochrane CENTRAL with the search terms (lung cancer or lung adenocarcinoma) AND (gefitinib) AND (pemetrexed) on June 23, 2022. Additional eligible trials were acquired through searching the references of relevant published clinical trials and review articles.

### Study selection

Inclusion criteria included: (1) Participants: previously untreated lung cancer with mutated EGFR; (2) Interventions: gefitinib with or without pemetrexed-based chemotherapy; (3) Comparison: gefitinib versus gefitinib plus pemetrexed-based chemotherapy; (4) Outcomes: data on survival outcomes, responses, and tolerability were available. English language and registered trials were eligible. Two authors performed the literature search and study selection independently, and any discrepancies were reviewed by a third author and resolved by consensus.

### Outcome measures and data extraction

The primary outcome measures comprised OS and PFS. The secondary outcome was objective response rate (ORR), disease control rate (DCR), and discontinuation rate (DR). Data extraction was conducted by two authors independently and reviewed by a third author. Data regarding the first author, year of publication, study design, disease stage of patients, number of patients, EGFR mutations, therapeutic strategies, median PFS, and median OS were recorded.

### Statistical analysis

Data on OS and PFS were evaluated by hazard ratio (HR) with 95% confidence intervals (CIs). ORR and DCR data were assessed by odds ratio (OR) with 95% CIs. While DR data were measured by risk ratio (RR) with 95% CIs. Statistical analyses were performed using R Studio (version 1.4.1717, R Foundation for Statistical Computing). The “meta” package was used to perform the fixed effect model and random-effects model meta-analyses and tests for heterogeneity (*I*^2^ and τ) [14]. A fixed effect model was selected over a random-effects model if *I* ≤ 50%. τ^2^ = 0 indicated that no deviations were found across the trials. Additionally, publication bias was evaluated by Egger’s tests and p > 0.01 indicated no publication bias.

## Results

### Eligible studies and characteristics

**Fig 1:**
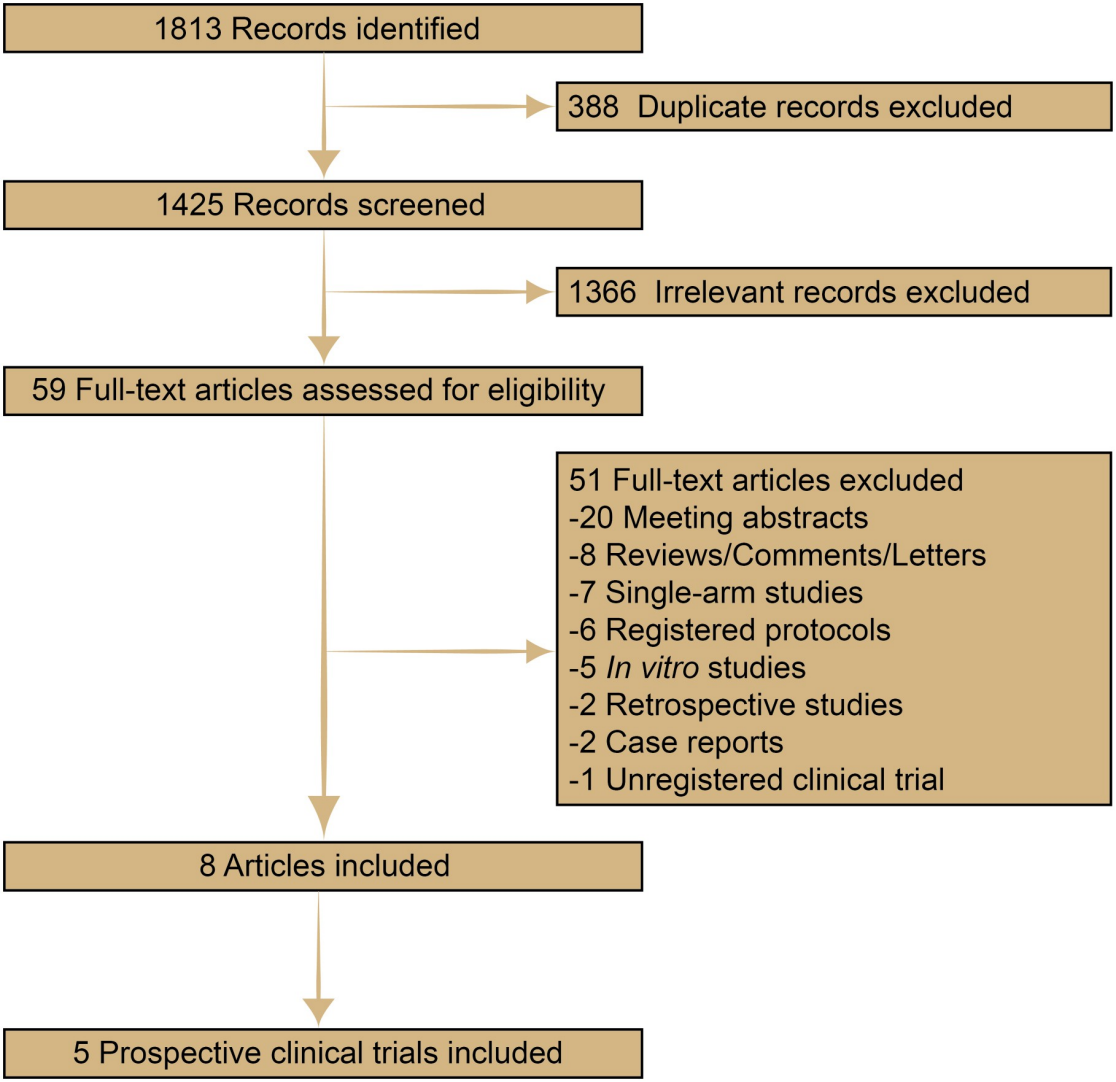
Flow chart of the selecting process.

1813 records were collected through a literature search and review of reference lists. After screening and eligibility evaluation, five registered, prospective, randomized clinical trials involving 1014 patients were included in the pooled analysis (Fig 1). The trials were reported between 2014 and 2020, as shown in Table 1 [8–10, 15–19]. Two were phase 2 trials, and the other three were phase 3 trials. Stage IV and stage IIIB patients who were not amenable to radical therapy were enrolled. In Noronha’s trials, two squamous NSCLC patients were involved. EGFR mutations comprised exon 19 deletion, exon 21 L858R, exon 21 S768I, exon 21 G719A, exon 21 G719C, exon 21 G719S, exon 21 L861Q, exon 18 G719X, exon 20 T790M, etc. Pemetrexed-based chemotherapeutic strategies included pemetrexed alone and pemetrexed plus carboplatin/cisplatin. In the gefitinib group, median OS ranged from 17.0 to 45.7 months, and median PFS ranged from 8.0 to 16.6 months. While in the gefitinib plus pemetrexed-based chemotherapy group, median OS ranged from 32.4 to 50.9 months, and median PFS ranged from 16.0 to 20.9 months.

**Table 1:** Basic characteristics of enrolled trials and survival outcomes.

First author	Year of Publication	Design	Patient stage	Number of Patients	Mutations	G	G+C	Median PFS (months)	Median OS (months)
Yang	2014/2016	A multicenter, open-label, randomized, phase 3 trial	Stage IIIB (T4-malignant pleural effusion) or stage IV non-squamous NSCLC	G+C: 26	EGFR exon19 deletion	250 mg/day	Pemetrexed 500 mg/m^2^ plus cisplatin 75 mg/m^2^ on day 1, every 3 weeks, up to 6 cycles, then non-progressing patients received oral gefitinib as maintenance therapy;	G+C: 12.9	G+C: 32.4 (19.3–NE)
					EGFR exon21 L858R, S768I				
		NCT01017874		G: 24	Other		Gefitinib 250 mg/day.	G: 16.6	G: 45.7 (25.8–NE)
Cheng/Yang	2016/2020	A multicenter, open-label, randomized, phase 2 trial	Stage IV non-squamous NSCLC	G+C: 126	EGFR exon19 deletion	250 mg/day	Pemetrexed 500 mg/m^2^ on day 1, every 3 weeks;	G+C: 16.2 (12.6–18.7)	G+C: 43.3 (33.4–50.8)
		NCT01469000		G: 65	EGFR exon21 L858R		Gefitinib 250 mg/day.	G: 11.1 (9.7–13.8)	G: 36.8 (26.7–42.6)
Han/Lou	2017/2020	A single-center, open-label, randomized, phase 2 trial	Locally advanced or metastatic adenocarcinoma (Stage IIIB or IV)	G+C: 40	EGFR exon19 deletion	250 mg/day	Pemetrexed 500 mg/m^2^ plus carboplatin AUC 5 on day 1, every 4 weeks, up to 6 cycles, then continued to receive pemetrexed every 4 weeks;	G+C: 17.5 (15.3–19.7)	G+C: 37.9 (17.3–58.6)
		NCT02148380		G: 41	EGFR exon21 L858R		Gefitinib 250 mg/day on days 5–21, every 4 weeks.	G: 11.9 (9.1–14.6)	G: 25.8 (19.2–32.3)
Hosomi	2020	A multicenter, open-label, randomized, phase 3 trial	Stage IIIB or IV or relapsed non-squamous NSCLC	G+C: 170	EGFR exon19 deletion, EGFR exon21 L858R, G719A, G719C, G719S, and L861Q	250 mg/day	Pemetrexed 500 mg/m^2^ plus carboplatin AUC 5 on day 1, every 3 weeks, up to 6 cycles, followed by concurrent gefitinib and pemetrexed maintenance;	G+C: 20.9 (17.9–24.2)	G+C: 50.9 (41.8–62.5)
		UMIN000006340		G: 172			Gefitinib 250 mg/day.	G: 11.7 (9.0–13.4)	G: 38.80 (31.1–47.3)
Noronha	2020	A single-center, open-label, randomized, phase 3 trial	Locally advanced stage IIIB NSCLC not amenable to radical therapy or stage IV NSCLC	G+C: 174	EGFR exon19 deletion	250 mg/day	Pemetrexed 500 mg/m^2^ plus carboplatin AUC 5 on day 1, every 3 weeks, up to 6 cycles, then continued to receive pemetrexed every 3 weeks;	G+C: 16.0 (13.5–18.5)	G+C: Not reached
					EGFR exon21 L858R/L861Q				
		CTRI/2016/08/007149		G: 176	EGFR exon18 G719X		Gefitinib 250 mg/day.	G: 8.0 (7.0–9.0)	G: 17.0 (13.5–20.5)
					EGFR exon20 T790M				

Abbreviations: NSCLC, non-small cell lung cancer; G+C, gefitinib plus pemetrexed-based chemotherapy; G, gefitinib; EGFR, epidermal growth factor receptor; PFS, progression-free survival; OS, overall survival.

### Overall survival

**Fig 2:**
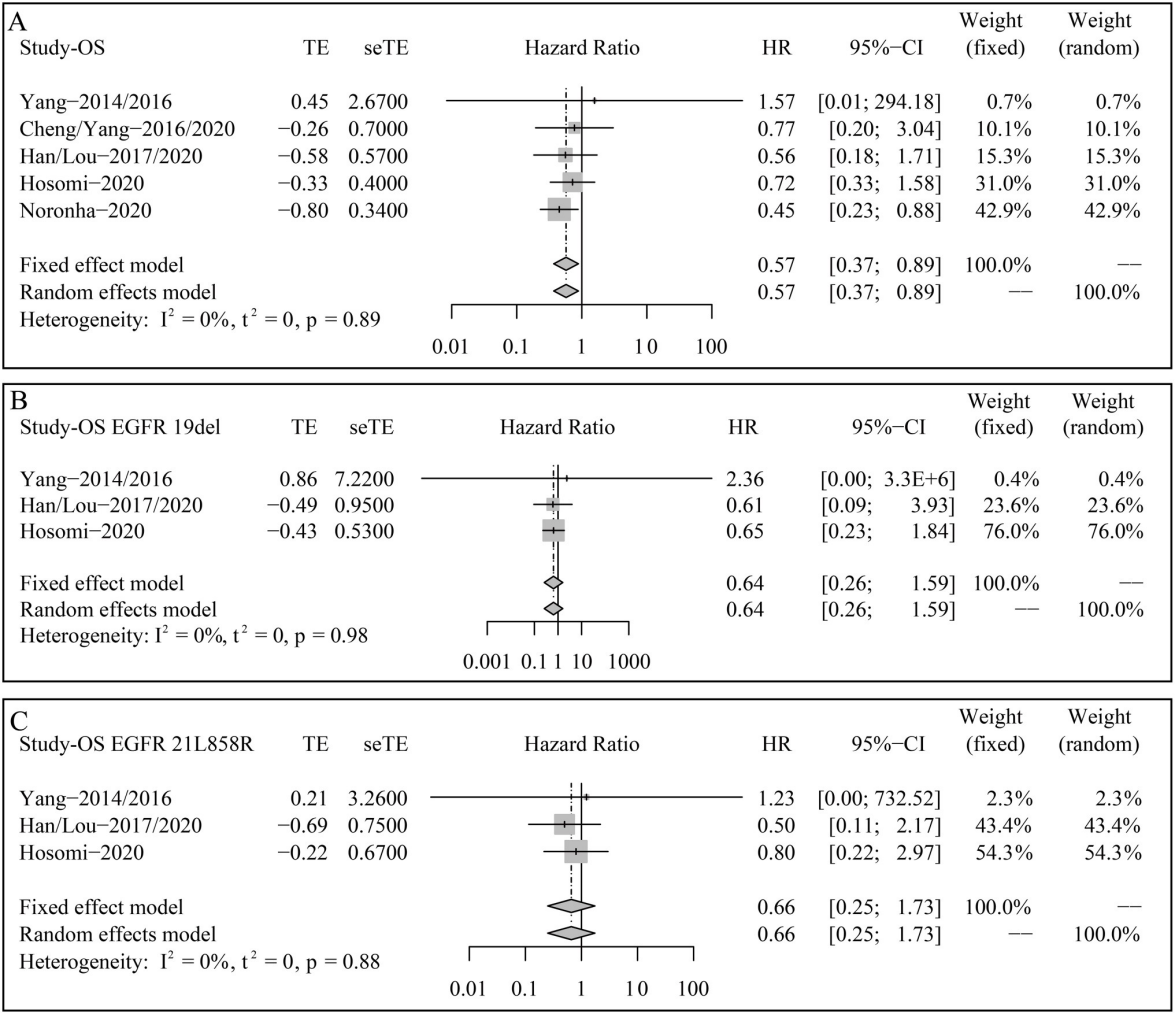
Forest plots of overall survival (OS) for hazard ratios (HRs). (A) Total enrolled patients; (B) Patients with EGFR exon 19 deletion; (C) Patients with EGFR exon 21 L858R.

Fig 2 depicts the forest plots of OS for HR. The estimated HR between gefitinib plus pemetrexed-based chemotherapy and gefitinib alone was 0.57 (95% CI 0.37–0.89, p = 0.0125; Heterogeneity: I^2^ = 0%; Fixed effect model), indicating that gefitinib plus pemetrexed-based chemotherapy significantly improved OS compared with gefitinib alone (Fig 2A).

In subgroup analysis, data from three trials were available. However, the addition of pemetrexed-based chemotherapy failed to decrease the risk of death in patients with EGFR exon 19 deletion (HR 0.64, 95% CI 0.26–1.59, p = 0.3402; Heterogeneity: I^2^ = 0%; Fixed effect model) and exon 21 L858R (HR 0.66, 95% CI 0.25–1.73, p = 0.3984; Heterogeneity: I^2^ = 0%; Fixed effect model) (Fig 2B and 2C).

### Progression-free survival

**Fig 3:**
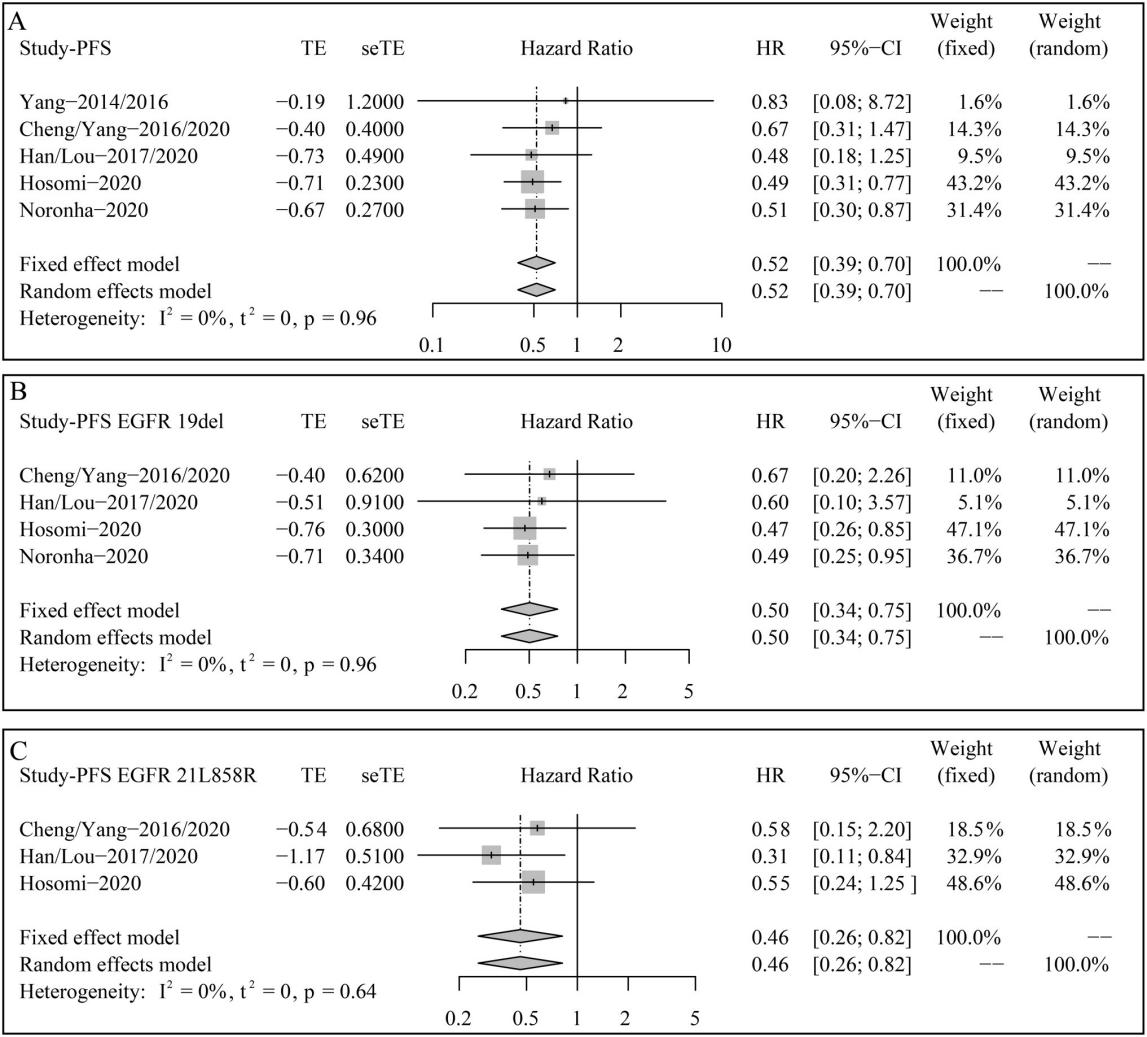
Forest plots of progression-free survival (PFS) for hazard ratios (HRs). (A) Total enrolled patients; (B) Patients with EGFR exon 19 deletion; (C) Patients with EGFR exon 21 L858R.

Fig 3 depicts the forest plots of PFS for HR. The estimated HR between gefitinib plus pemetrexed-based chemotherapy and gefitinib alone was 0.52 (95% CI 0.39–0.70, p < 0.0001; Heterogeneity: I^2^ = 0%; Fixed effect model), demonstrating that combination of gefitinib and pemetrexed-based chemotherapy significantly prolonged PFS versus gefitinib (Fig 3A).

Similarly, subgroup analyses found that the adding pemetrexed-based chemotherapy to gefitinib significantly decreased the risk of disease progression or death in patients with EGFR exon 19 deletion (HR 0.50, 95% CI 0.34–0.57, p = 0.0008; Heterogeneity: I^2^ = 0%; Fixed effect model) and exon 21 L858R (HR 0.46, 95% CI 0.26–0.82, p = 0.0079; Heterogeneity: I^2^ = 0%; Fixed effect model) (Fig 3B and 3C).

### Responses

**Fig 4:**
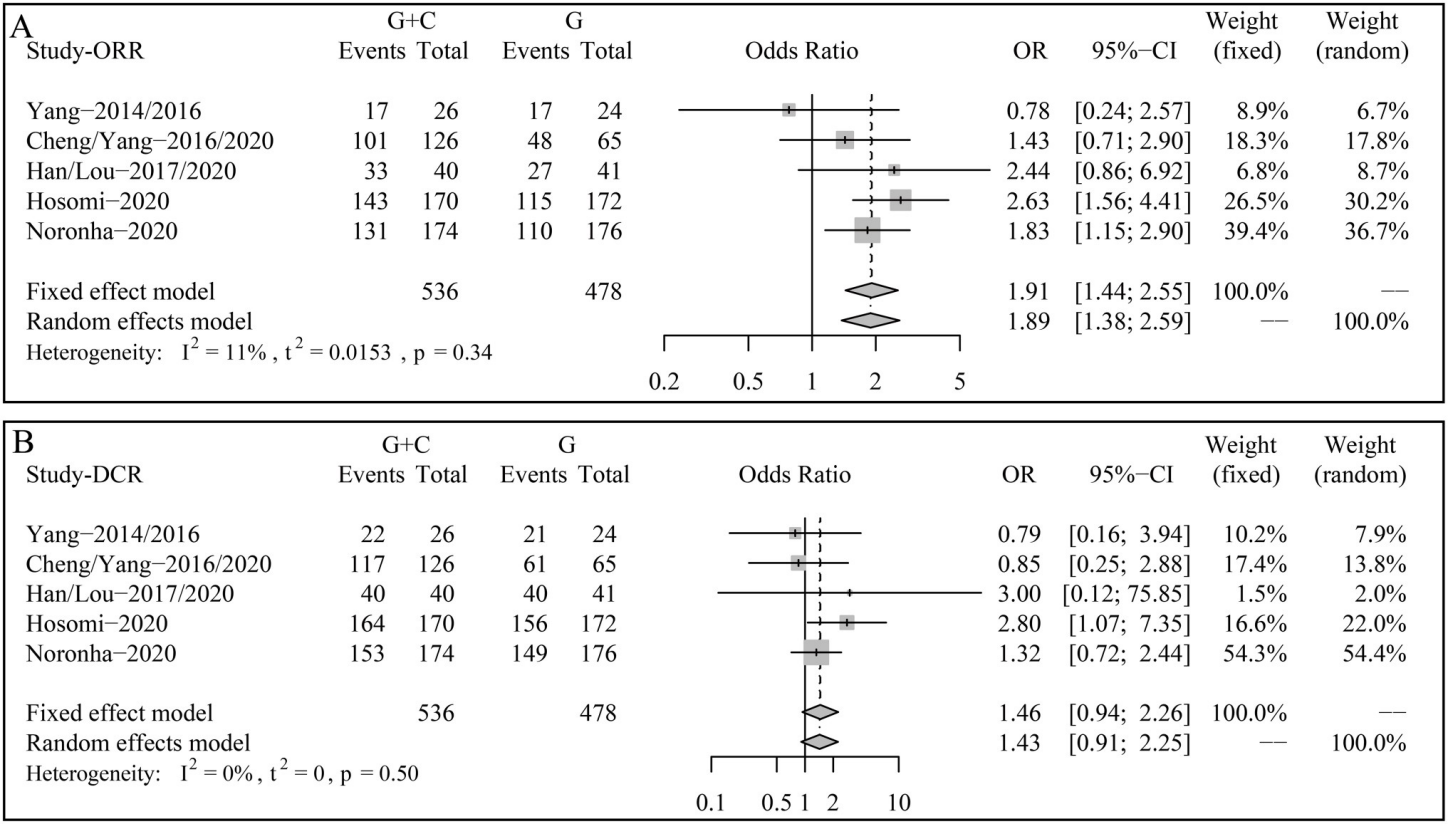
Forest plots of (A) objective response rate (ORR) and (B) disease control rate (DCR) for odds ratios (ORs).

All patients were eligible for the analyses of ORR and DCR. Compared with gefitinib monotherapy, adding pemetrexed-based chemotherapy to gefitinib showed a 1.91-time ORR (OR 1.91, 95% CI 1.44–2.55, p < 0.0001; Heterogeneity: I^2^ = 11%; Fixed effect model) (Fig 4A). Nevertheless, in terms of DCR, the difference between the two groups was not statistically significant (OR 1.46, 95% CI 0.94–2.26, p = 0.0952; Heterogeneity: I^2^ = 0%; Fixed effect model) (Fig 4B).

### Discontinuation rate

**Fig 5:**
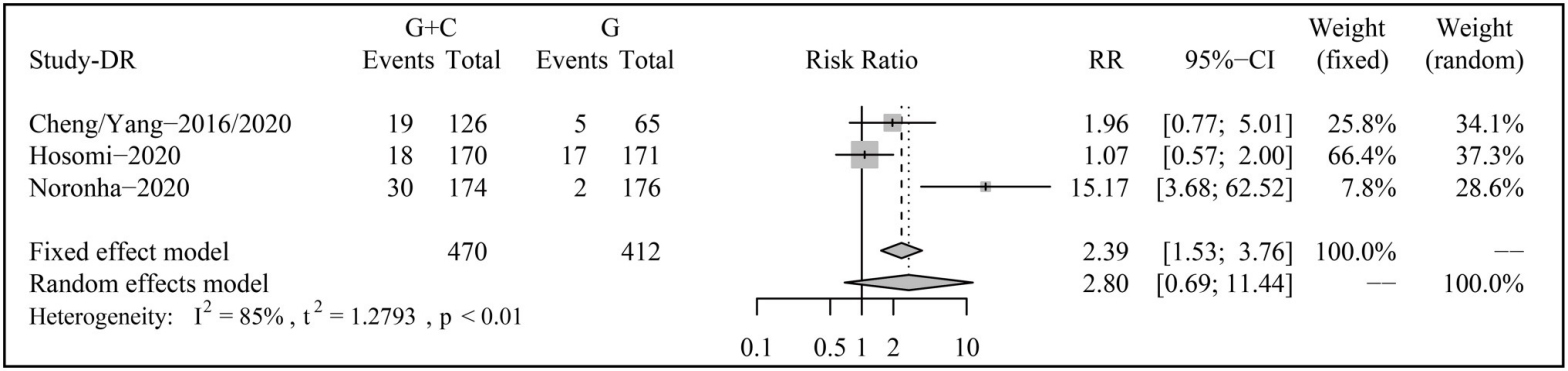
Forest plots of discontinuation rate (DR) for risk ratios (RRs).

The tolerability was assessed by DRs in this study (Fig 5). Three trials involving 470 patients in the gefitinib plus pemetrexed-based chemotherapy group and 412 patients in the gefitinib group. Although the DR was higher in the combination therapy against monotherapy, no statistical differences were found (RR 2.80, 95% CI 0.69–11.44, p = 0.1509; Heterogeneity: I^2^ = 85%; Random-effects model).

### Publication bias

**Fig 6:**
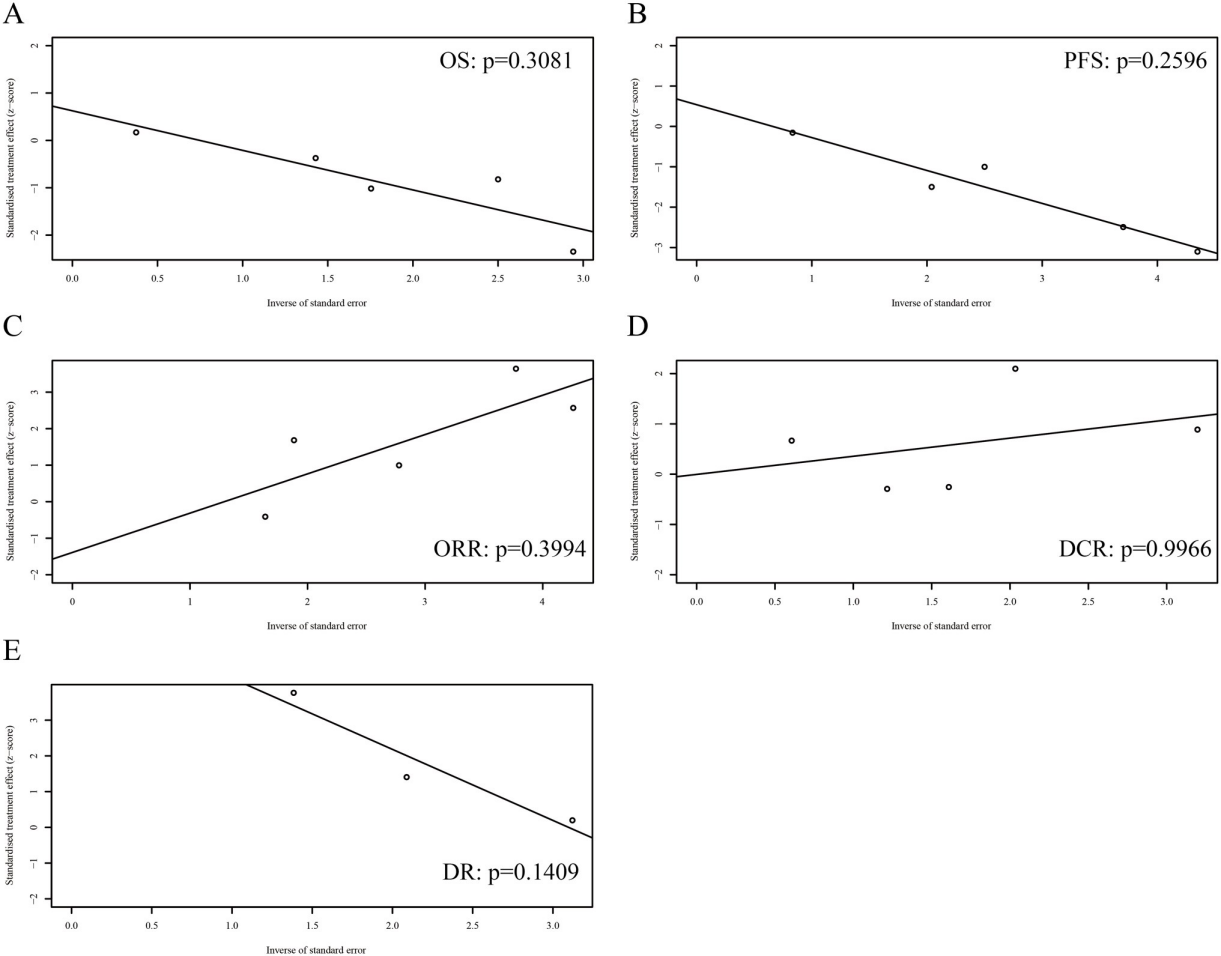
Egger’s tests for (A) overall survival (OS), (B) progression-free survival (PFS), (C) objective response rate (ORR), (D) disease control rate (DCR), and (E) discontinuation rate (DR).

Fig 6 shows the Egger’s tests in the analyses of OS (p = 0.3081), PFS (p = 0.2596), ORR (p = 0.3994), DCR (p = 0.9966), and DR (p = 0.1409), indicating the absence of publication bias.

## Discussion

We performed a pooled analysis of gefitinib with or without pemetrexed-based chemotherapy in treating advanced previously untreated EGFR mutation-positive NSCLC patients. The combination of gefitinib and pemetrexed-based chemotherapy was found to be superior to gefitinib monotherapy, with improved OS, PFS, and ORR. However, OS benefits were not reported in patients with EGFR exon 19 deletion or exon 20 L858R.

To enhance the effects and conquer the resistance of TKIs, clinicians have tried various therapeutic forms in the first-line treatment for advanced NSCLC patients with mutated EGFR.

The FLAURA trial has provided solid evidence of the superiority of osimertinib versus first-generation TKI in EGFR mutation-positive advanced NSCLC [20, 21]. The median PFS was 18.9 months in the osimertinib group versus 10.2 months in the first-generation TKI group (HR 0.46, 95% CI 0.37–0.57, p < 0.001) [20]. The median OS was 38.6 months versus 31.8 months (HR 0.80. 95% CI 0.64–1.00, p 0.046) [21]. In the FLAURA2 trial, osimertinib plus pemetrexed and platinum chemotherapy was administered to achieve a higher efficacy and longer survival. The safety run-in results reported a manageable safety and tolerability profile for the combination therapy [22]. In detail, the most common treatment-related adverse event was constipation (60%), and 20% of enrolled patients suffered serious treatment-related adverse events [22]. Future results may uncover the feasibility, efficacy, and safety of combining third-generation EGFR TKI and pemetrexed-based chemotherapy.

As the application of third-generation EGFR TKI, the benefits from first-generation EFGR TKI monotherapy or combined with chemotherapy remains controversial. However, once NSCLC patients have got resistant to third-generation TKI therapy, no other effective targeted therapeutic drugs can be administered. This is a critical point that should not be ignored. Nevertheless, third-generation TKI therapy could be an optimal option for EGFR mutation-positive NSCLC patients after first-generation TKI therapy. Therefore, first-line TKI-based therapies are meaningful. Additionally, in treating advanced NSCLC patients with mutated EGFR, only comparing different drugs in one line treatment may not be enough. Making long-range therapeutic planning might be more practical for advanced EGFR-mutated NSCLC patients in real-world clinical practice.

Antiangiogenic therapy could be an option for a subset of patients who are not well tolerated in pemetrexed-based chemotherapy. In Huang’s study, different EGFR TKIs (including gefitinib, erlotinib, and afatinib) plus bevacizumab strategies were investigated as the first-line treatment [23]. The median PFS was 16.4 months with a 77.7% ORR and a 94.4% DCR. Moreover, longer PFS was observed in patients with brain metastasis at baseline (erlotinib: 18.9 months; afatinib: 16.4 months) [23]. In another retrospective study, first-line EGFR TKI combined with bevacizumab was compared with EGFR TKI monotherapy in patients with EGFR-sensitive mutant NSCLC (31982639). The DCR (95% versus 74.2%) and PFS (16.5 months versus 12.0 months) were significantly improved, with an acceptable safety profile.

Palliative radiotherapy might be an effective treatment for advanced NSCLC patients with EGFR mutations. The radiation sites could be primary lung tumors or brain or bone metastatic lesions. Hou reported an NSCLC patient with bone metastasis with more than eight-year survival. For this patient, the primary lung tumor (62 Gy/31 fractions) and lumbar spinal metastatic lesions (50 Gy/25 fractions) received local radiotherapy [24]. In a randomized clinical trial, newly diagnosed advanced EGFR-mutant NSCLC patients were treated with a first-generation TKI with or without primary and metastases radiotherapy, revealing that up-front local radiotherapy significantly prolonged PFS (20.2 months versus 12.5 months) and OS (25.5 months versus 17.4 months) [25]. In another circumstance where patients had bone metastases after establishing clinical effects from EGFR TKI therapy, radiotherapy for the bone lesions could make the continuous administration of EGFR TKI therapy possible [26]. The prognosis remains poor for patients with brain metastasis, even if radiotherapy (whole-brain radiation therapy or stereotactic radiosurgery) is administered [27]. However, in Miyawaki’s study, patients with 1–4 brain metastases were found to benefit more from the local radiotherapy plus TKI than TKI monotherapy (OS: 35 months versus 23 months; HR 0.54, 95% CI 0.32–0.90) [28].

Several limitations should be mentioned. First, different chemotherapeutic strategies were included. For example, platinum drugs used in the trials included cisplatin and carboplatin; pemetrexed plus cisplatin/carboplatin with or without pemetrexed maintenance therapy. Although deviations across the trials existed, they were low and might not have much impact on our analysis. Second, treatment-related adverse events were not pooled-analyzed since the toxicities during both gefitinib and gefitinib plus pemetrexed-based chemotherapy are well-known and manageable. The most comment treatment-related adverse events comprised leucopenia, neutropenia, anemia, alanine transaminase increased, aspartate transaminase increased, and fatigue. Third, subgroup analysis only included data of EGFR exon 19 deletion and exon 21 L858R. More detailed explorations of the efficacy of gefitinib on exon 20 S78I, exon 21 L861Q, exon 18 G719X, and even exon 20 T790M mutations are needed in future studies.

## Conclusions

The addition of pemetrexed-based chemotherapy to gefitinib could be administered as the first-line therapy for advanced EGFR-mutant NSCLC patients. Future studies may lead to explore more subtypes of EGFR mutations suitable for receiving a TKI plus pemetrexed-based chemotherapy.

## Supporting information

S1 Checklist (DOC)
